# 

**Published:** 1795

**Authors:** 


### CONTENTS.

Page

I. *OBSERVATIONS on the Use of Arsenic in the Intermittent Fevers of a tropical Climate; to which is prefixed an Account of the Weather, at Sierra Leone, during the Season in which such Fevers are most prevalent. By* Thomas Masterman Winterbottom, *M. D. Physician to the Settlement at Sierra Leone.* — — 1

II. *An Account of the good Effects of a Solution of Sal Ammoniac, in Vinegar, employed, as a topical Application, in Cases of lacerated Wounds. By Mr.* Henry Yates Carter, *Surgeon at Kettley, near Wellington, in Shropshire.* — — 66

III. *Case of a diseased Kidney. By the same.* 85

IV. *Case of a Gun-Shot Wound of the Head. By the same.* — — — 91

V. *An Account of some extraordinary Symptoms which were apparently connected*

with

Page

*with certain morbid Alterations about the Veins and Nerves. By Mr.* John Pearſon, *Surgeon of the Lock Hoſpital, and of the Public Diſpenſary.* — 96

VI. *An Account of the Extraction of an extraneous Subſtance from the Rectum. By Mr.* William Blair, *Surgeon of the Lock Hoſpital; and of the General Diſpenſary in Newman Street, St. Mary-le-bone.* 111

VII. *A Caſe of Aneuriſm of the Crural Artery. By Mr.* Thompſon Forſter, *Surgeon on the Staff of the Army, and Surgeon to Guy's Hoſpital.* —— — 114

VIII. *An Account of a Key Inſtrument of a new Conſtruction; with Obſervations on the Principles on which it acts, in the Extraction of Teeth, and on the Mode of applying it. By Mr.* Robert Clarke, *Surgeon at Sunderland, in the County of Durham.* 120

IX. *An Account of a new Species of Swietenia (Mahogany); and of Experiments and Obſervations on its Bark, made with a View to aſcertain its Powers, and to compare them with thoſe of Peruvian Bark, for which it is propoſed as a Subſtitute:*

Page

*Being an Abstract of a Paper on this Subject, addressed to the Honourable Court of Directors of the United East-India Company. By* William Roxburgh, *M.D.* 127

X. *An Account of the Effects of Mahogany Wood in Cases of Diarrhœa. By Mr.* Francis Hughes, *Surgeon of the General Infirmary at Stafford.* — — 156

XI. *Account of some Discoveries made by Mr.* Galvani, *of Bologna; with Experiments and Observations on them. In two Letters from Mr.* Alexander Volta, *F.R.S. Professor of Natural Philosophy in the University of Pavia, to Mr.* Tiberius Cavallo, *F. R. S.—From the Philosophical Transactions of the Royal Society of London.* — — — 162

XII. *A Return of the Sick of the Ship's Company, and of the Military, on Board the Ships in the Service of the Honourable the United East-India Company, for the Years* 1792 *and* 1793. *By* John Lorimer, *M. D.* — — 211

XIII. *An Account of a singular Case of Ischuria, in a young Woman, which continued*

Page

*for more than three Years; during which Time, if her Urine was not drawn off with the Catheter, ſhe frequently voided it by vomiting; and, for the laſt twenty Months, paſſed much Gravel by the Catheter, as well as by vomiting, when the Uſe of that Inſtrument was omitted, or unſucceſsfully applied. To which are added ſome Remarks and Phyſiological Obſervations. By* Iſaac Senter, *M. D. Aſſociate Member of the College of Phyſicians of Philadelphia, and ſenior Surgeon in the late American Army.* Vide *Tranſactions of the College of Phyſicians, of Philadelphia.* — — 212

*Catalogue of Books.* — — 223

*Index.* — — — 228

